# Cell-Cultured Influenza Vaccine Enhances IFN-γ+ T Cell and Memory T Cell Responses Following A/Victoria/2570/2019 IVR-215 (A/H1N1) Infection

**DOI:** 10.3390/vaccines12121392

**Published:** 2024-12-11

**Authors:** Kyu-Ri Kang, Pan-Kyeom Kim, Kyung-Min Jo, Jin-Young Jang, Hyun Mi Kang, Jin-Han Kang

**Affiliations:** 1 The Vaccine Bio Research Institute, College of Medicine, The Catholic University of Korea, Annex to Seoul Saint Mary Hospital, 222 Banpo-daero, Seocho-gu, Seoul 06591, Republic of Korea; krk@vric.kr; 2 SK Bioscience, 332 Pangyo-ro, Bundang-gu, Seongnam-si 13493, Republic of Korea; 4u4us@sk.com (P.-K.K.); fluvrd@sk.com (K.-M.J.); jinyoung421@sk.com (J.-Y.J.); 3 Department of Pediatrics, College of Medicine, The Catholic University of Korea, 222 Banpo-daero, Seocho-gu, Seoul 06591, Republic of Korea

**Keywords:** T cells in influenza vaccine immunity, cell-cultured influenza vaccine, egg-based influenza vaccine, influenza vaccine efficacy, immune cells in influenza vaccine immunity

## Abstract

Background: Influenza remains a significant public health challenge, with vaccination being a substantial way to prevent it. Cell-cultured influenza vaccines have emerged to improve on the drawbacks of egg-based vaccines, but there are few studies focusing on T cell immunity with both types of vaccines. Therefore, we studied the following 2022–2023 seasonal influenza vaccines with a standard dose and high dose: cell-based (C_sd and C_hd) and egg-based (E_sd and E_hd) vaccines. Methods: Along with a saline control group, C_sd, C_hd, E_sd, and E_hd vaccines were administered to BALB/c mice, followed by a challenge with the A/Victoria/2570/2019 (H1N1) strain. Results: After the challenge, four out of five mice in the saline group died by day 7 post-infection (P.I.). None of the vaccinated groups experienced over 20% weight loss or any deaths. On day 7 P.I., the lung viral load in the saline group (mean log value of 4.17) was higher than that in the vaccinated groups, with the C_sd group showing the lowest viral load (mean log value of 3.47). The C_sd group showed a significantly high response in macrophage 1 (M1), IFN-γ+ T cells, and tissue-resident memory (T_RM_) T cells compared with the E_sd group on day 2 P.I. These M1, IFN-γ+ T cells, and T_RM_ cells showed similar trends (*p* < 0.01). In terms of humoral immunity, only the E_hd group showed HAI titers above 40 for all four strains before and after the challenge. Conclusions: The high levels of T cells in the cell-cultured vaccines suggest, pending further real-world research, that these vaccines may offer advantages.

## 1. Introduction

Influenza is an acute respiratory illness caused by influenza viruses. It remains a significant public health concern due to its ability to cause widespread illness and serious outcomes during seasonal outbreaks, as well as high mortality during pandemics. The 1918 H1N1 pandemic, for instance, resulted in an estimated 50 to 100 million deaths, while the 2009 pandemic claimed at least 200,000 lives [1,2]. Despite a decrease in the fatality rate of influenza over time, the ongoing antigenic shift and the potential for antigenic drift in the virus continue to present a significant threat of future pandemics [3]. Although influenza typically manifests as a mild febrile illness, it can progress to severe disease or result in complications, particularly in high-risk populations such as the elderly, young children, and individuals with underlying chronic conditions or compromised immune systems [4,5,6]. Vaccination remains the most effective and cost-efficient strategy for preventing influenza [7], with both inactivated and live attenuated vaccines currently approved for use.

Influenza vaccines have traditionally been produced using fertilized chicken eggs; however, this approach presents several significant limitations, including prolonged production timelines, the risk of egg protein allergies, the occurrence of hemagglutinin (HA) mutations, and difficulties in achieving large-scale production [8]. To overcome these limitations, cell line-based vaccines have been developed as a next-generation alternative, with the first of these vaccines receiving approval in the United States in 2016 for the H3N2 strain [9]. The benefits of this approach were particularly evident during the 2017–2018 influenza season, when antigenic drift compromised the effectiveness of the egg-based H3N2 vaccine [10,11].

In South Korea, SK Bioscience markets the cell line-based seasonal influenza vaccine, SKYCellflu^®^, and both cell-cultured and egg-cultivated influenza vaccines are currently produced and commercially available. Cell cultured vaccines utilize a subunit platform and generally, egg-based vaccine is splitting platform, the immunological differences arising from their distinct production methods have not been extensively studied. Meanwhile, globally, numerous studies on the immunogenicity of cell-cultured versus egg-cultivated vaccines have shown mixed results: some studies found no significant differences between the two vaccines [12,13], while others suggested that the cell-cultured vaccine has immunological advantages [14,15]. Particularly, among these many studies, we found that there were few comparative studies focusing on T cells and other immune cells. In influenza immunogenicity, T cells are very important, as CD4+ and CD8+ T cells respond to conserved viral proteins, allowing them to recognize and react to multiple influenza strains [16,17]. Additionally, tissue-resident memory (T_RM_) CD4+ and CD8+ T cells, which persist in lung tissue, have been shown to enhance protective immune responses during influenza infection [18].

Consequently, this study seeks to compare the humoral and T cell immune responses elicited by cell-based and egg-based vaccines in a mouse model and to compare other immune cell responses. By conducting these comparisons, we aim to gain a deeper understanding of the mechanisms through which each vaccine confers protection against the influenza virus.

## 2. Materials and Methods

### 2.1. Vaccination and Challenges

In this study, the MDCK (Madin–Darby canine kidney) cell-based vaccine, SKYCellflu^®^ (SK bioscience, Bundang, Republic of Korea), and an egg-based vaccine were used. Both vaccines included the recommended viruses for use in the 2022–2023 season in the Northern Hemisphere: A/Victoria/2570/2019 (A/H1N1), A/Darwin/9/2021 (A/H3N2), B/Austria/1359417/2021 (B Victoria lineage), and B/Phuket/3073/2013 (B Yamagata lineage). Each vaccine contained 15 µg of hemagglutinin (HA) per virus strain in 0.5 mL of sterile water for injection. Normal saline was injected as a control. 

Five groups of 4-week-old female BALB/c mice (10 mice per group) were studied: a saline control group, egg-based vaccine standard-dose (E_sd) and high-dose (E_hd) groups, and cell-based vaccine standard-dose (C_sd) and high-dose (C_hd) groups. All mice were housed in pathogen-free conditions, and animal procedures were performed in accordance with the Institutional Animal Care and Use Committee (IACUC) guidelines and approved by the IACUC of the Catholic University of Korea (approval number: CUMS-2023-0167-04). 

**Figure 1:**
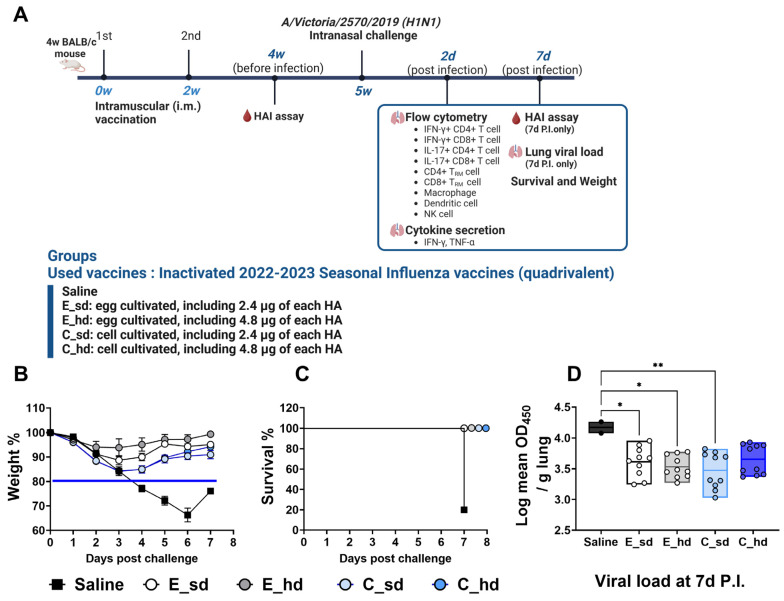
Weight loss and viral titer in lungs. Three weeks after the second vaccination, mice were challenged intranasally with 50 µL of A/Victoria/2570/2019 IVR-215 (A/H1N1) virus (768 HA titer). Five mice were tested per group. (**A**) The scheme of the study is depicted using the BioRender program (Toronto, ON, Canada). (**B**) Body weight was monitored daily, and changes were expressed as a percentage relative to the pre-vaccination weight. The blue line indicates a humane endpoint of 20%. (**C**) Survival and symptoms were observed daily for 7 days post-infection. Survival significance thresholds were calculated using the log-rank (Mantel–Cox) test. (**D**) On day 7 post-infection, the lung viral titer was evaluated with the influenza NP antibody, and OD_450_ values were log-transformed and presented as mean ± SEM (standard error of the mean). Each group had 10 pieces of data, since a dilution factor of 2 (1:100, 1:1000) was used in all groups except for the saline group at 7 days P.I. (n = 1), when 2 pieces of data were used. Differences between groups in the lung viral titer were determined by one-way ANOVA with Tukey’s multiple comparison tests, with *p*-values indicated as * *p* < 0.05, ** *p* < 0.01.

The standard-dose group received 2.4 µg HA per 4 strains in 80 µL of the cell-based or egg-based vaccine, and the high-dose group received 4.8 µg HA per 4 strains in 160 µL of the vaccine, which was injected into both thigh muscles. Each vaccine group received 2 doses at a 2-week interval (week 0 and week 2). Two weeks after the second vaccine dose (week 4), blood was collected via orbital puncture for the pre-infection HAI assay. Three weeks after the second dose, all mice were anesthetized with Zoletil 50^®^ Inj. (Virbac, Carros, France) and Rompun^®^ Inj. (Bayer, Leverkusen, Germany) and then infected intranasally with 50 µL of A/Victoria/2570/2019 virus at an LD_50_ dose of 768 HA titer. Blood and lung samples were collected on day 2 and day 7 post-infection (P.I.), and body weight and survival were monitored for 7 days P.I. (Figure 1A). 

### 2.2. Weight Loss, Survival, and Viral Titer

After a single challenge with the Madin–Darby canine kidney (MDCK) (CCL-24, ATCC, USA) cells passaged in the A/Victoria/2570/2019 IVR-215 (A/H1N1) virus at an LD_50_ dose corresponding to a hemagglutination (HA) titer of 768, the body weight changes and survival of the mice were monitored daily for 7 days, with a body weight change of more than 20% considered the humane endpoint. For the viral titer assay, we modified a previous method [19]. MDCK cells were cultured with high-glucose DMEM (Dulbecco’s Modified Eagle Medium) with L-glutamine and without sodium pyruvate (SH30022.01, HyClone Laboratories, Logan, UT, USA), and with 10% fetal bovine serum (FBS; Invitrogen Life Technologies), penicillin (100 U/mL), and streptomycin (100 µg/mL). On day 7 P.I., 2 lobes of the lungs were placed in DPBS containing a protease inhibitor cocktail (Roche cOmplete™, Roche, Basel, Switzerland), homogenized, and centrifuged. The supernatant was diluted by a factor of 10 and cultured for 2 days with MDCK cells in DMEM (Invitrogen Life Technologies, Carlsbad, CA, USA) supplemented with 10% fetal bovine serum (FBS; Invitrogen Life Technologies), 2 mM L-glutamine, 1 mM sodium pyruvate, penicillin (100 U/mL), streptomycin (100 µg/mL), and TPCK-treated trypsin at 0.1 µg/mL. The influenza A NP antibody (Merck MAB 8257, Merck MAB 8258) was used as a primary antibody and anti-mouse IgG H&L HRP-conjugated antibody (5450-0011, KPL, LGC Clinical Diagnostics, Milford, MA, USA) was used as a secondary antibody. The cytopathic effect (CPE) in the lungs due to the virus was assessed by measuring OD_450_ after adding antibodies, and the results were normalized to lung weight.

### 2.3. T Cell and Other Immune Cell Responses

At 2 days and 7 days P.I. with the A/Victoria/2570/2019 IVR-215 (A/H1N1) virus, mice were anesthetized with Zoletil 50^®^ Inj. (Virbac, Carros, France) and Rompun^®^ Inj. (Bayer, Leverkusen, Germany), and lung tissue was collected to assess the expression of IFN- γ and IL-17 in CD8+ T cells and CD4+ T cells, as well as in CD4+ T_RM_ cells and CD8+ T_RM_ cells. The lung tissue was incubated for 30 min with 40 U/mL DNase I and 1 mg/mL Collagenase IV enzyme and then dissociated using a tissue dissociator and filtered through a 100 μM cell strainer. Then, RBCs were lysed to prepare cells at a concentration of 4 × 10^6^ cells/mL. The prepared cells were incubated with Brefeldin A (BD Bioscience, Franklin Lakes, NJ, USA) for over 6 h at 37 °C and then washed with PBS and blocked for Fc receptors using CD16/CD32 antibodies (BD Pharmingen Mouse Fc Block). T cells were stained for markers as shown for the T cells in Table 1. The cells were fixed and permeabilized using BD Cytofix/Cytoperm Plus (BD Bioscience) before intracellular cytokines were stained. The BD FACS Aria flow cytometer (BD Biosciences) was used to analyze 37,000 lung single cells per group on day 2 post-infection and 52,700 cells on day 7 post-infection. The results were analyzed using FlowJo (BD Biosciences) version 10.9.0. For the macrophage and for dendritic immune cell response, lung cells were prepared using the same method as for T cells, then washed, blocked for Fc receptors, and stained for markers as shown for the immune cells in Table 1 below. The cells were analyzed with the BD FACS Aria flow cytometer (BD Biosciences). The results were analyzed with FlowJo software.

### 2.4. Cytokine Secretion

After a single challenge with A/Victoria/2570/2019 IVR-215 (A/H1N1), lung single cells were prepared according to the method described in the T cell responses Section. Single lung cells from lung tissues on day 2 post-infection and day 7 post-infection were prepared at a concentration of 4 × 10^6^ cells/mL in a 96-well plate. An amount of 100 μL per well was used, and these were cultured for 3 days. The concentrations of IFN-γ and TNF-α in the culture supernatant were then measured using commercially available cytokine ELISA kits from Proteintech (Rosemont, IL, USA).

### 2.5. HAI Assay (Hemagglutinin Inhibition Assay)

Two weeks after the second vaccination and 7 days P.I., blood was collected from the mice at 2 time points, and serum was separated. Antigens for the 2022–2023 Northern Hemisphere seasonal influenza strains were purchased from NIBSC (Table 2), and the HAI assay was conducted following the established method [20]. Mouse serum was treated with receptor-destroying enzyme (RDE) from Vibrio cholera (Denka Seiken, Tokyo, Japan) at a ratio of 3 times the serum volume and incubated overnight at 37 °C. Following this, the serum was treated with 3 times the volume of 2.5% sodium citrate solution and incubated at 56 °C for 30 min. The serum was then diluted 3-fold with PBS, followed by a serial 2-fold dilution in a 96-well plate. The diluted serum was reacted with 4 hemagglutination (HA) units/25 µL of NIBSC antigen and 0.5% Turkey RBCs. The highest dilution at which hemagglutination was inhibited, resulting in no visible agglutination (a clear well), was recorded as the final titer of the serum.

### 2.6. Statistical Analysis

All results and graphs were generated using GraphPad Prism™ software v9 (GraphPad, San Diego, CA, USA). Comparisons between groups were made using one-way ANOVA with Tukey’s multiple comparison test or with the non-parametric Kruskal–Wallis multiple comparison test. Statistical significance was determined by *p*-values (* *p* < 0.05, ** *p* < 0.01, *** *p* < 0.001, **** *p* < 0.0001); *p*-values lower than 0.01 were considered significantly different between groups.

## 3. Results

### 3.1. The C_sd Group Showed Significantly Low Viral Titers Compared with the Saline Group 

On day 4 P.I., four out of the five mice in the saline group exhibited more than 20% body weight loss and showed signs of decreased activity. By day 5, all mice in this group had lost more than 20% of their body weight, and by day 7, four had died (Figure 1B,C). In contrast, the other vaccinated groups showed the greatest average body weight loss on day 3 P.I., but none of these groups exhibited an average body weight loss exceeding 20%. The mice in these groups gradually recovered, and there were no deaths. On day 5 P.I., the C_sd group showed a difference in the body weight change compared to the E_sd and E_hd groups (*p* < 0.05), but this difference was not statistically significant (Figure 1B). The lung viral load was assessed on day 7 P.I. by measuring the residual virus in lung homogenates, with the saline group showing a mean log value of 4.17, which was statistically significantly higher than in all the vaccinated groups. There were no significant differences among the vaccinated groups, with all showing similar values. The C_sd group had the lowest mean log value of 3.47 (Figure 1D) and demonstrated significant differences compared with the saline group.

### 3.2. The C_sd Group Showed a High IFN-γ+ T Cell and Memory T Cell Count on Day 2 Post-Infection

**Figure 2:**
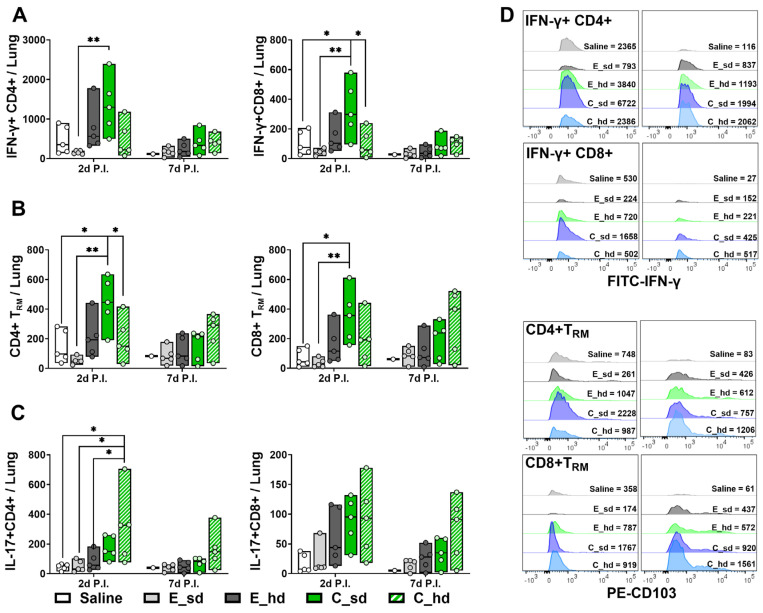
IFN-γ+ T cell, CD4+ T_RM_ cell, CD8+ T_RM_ cell, and IL-17+ T cell response. The absolute numbers of T cells expressing (**A**) IFN-γ+ CD8+ and IFN-γ+ CD4+ are shown with box plots. (**B**) CD8+ T_RM_ cell, CD4+ T_RM_ cell, and (**C**) IL-17+ T cell counts are also shown with box plots (mean cell count depicted by line in min to max box). (**D**) IFN-γ+ T cell and T _RM_ cell counts are depicted as histograms for the groups (n = 5, with 1 mouse in the saline group on day 7 P.I.). Differences between groups were determined by one-way ANOVA with Tukey’s multiple comparison test, with *p*-values indicated as * *p* < 0.05, ** *p* < 0.01.

T cells were analyzed with a total of 45,000 lung single cells per group on day 2 P.I. and a total of 73,404 lung single cells per group on day 7 P.I. The C_sd group had the highest IFN-γ+ CD4+, CD8+ T cell, and tissue-resident memory T cell amounts on day 2 P.I., and the amounts were statistically significantly different (*p* < 0.01) compared to the results of the E_sd group (Figure 2A,B,D). The C_hd group demonstrated the highest T cell responses on day 7 P.I., but no significant differences were observed between groups (Figure 2A,B,D). IL-17+ CD4+, CD8+ T cell showed dose dependent result and no significant differences between groups (Figure 2C and Figure S1).

### 3.3. The C_sd Group Showed a High Number of Macrophage 1 (M1) Cells on Day 2 Post-Infection

**Figure 3:**
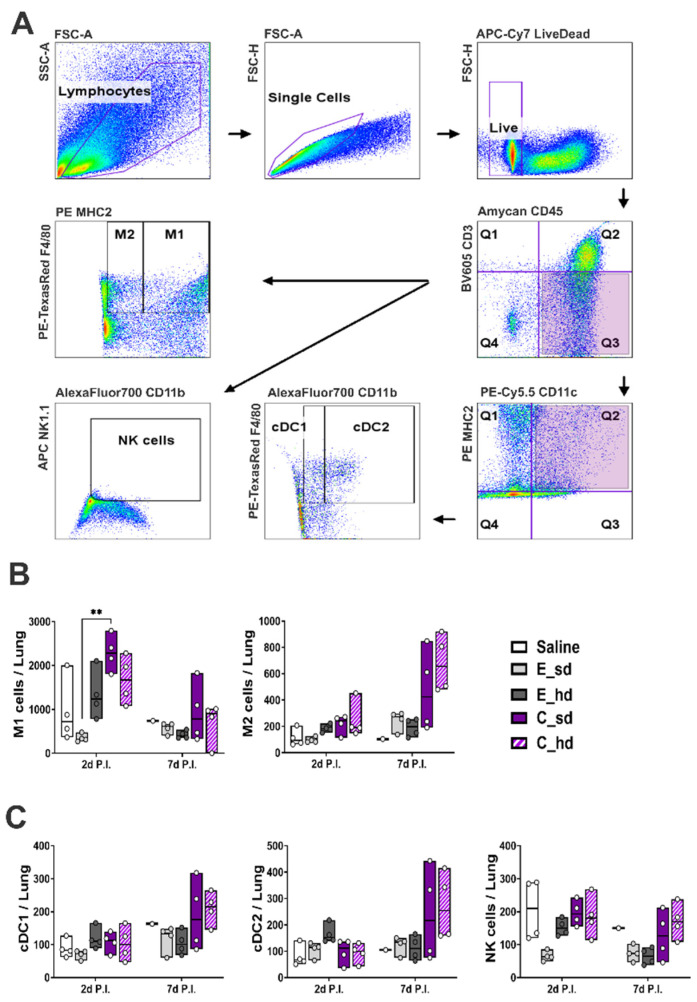
Macrophage, dendritic cell, and NK cell responses. (**A**) Gating strategies for macrophages, dendritic cells, and NK cells. Live single cells were gated based on size and granularity by FSC-A and FSC-H and by live cell marker. Macrophages, dendritic cells, and NK cells were gated with CD45+ CD3-. Then, dendritic cells were gated with CD11c + MHC2+. Conventional DC1 was gated with CD11b- F4 80+, and cDC2 was gated with CD11b+ F4 80+. NK cells were defined with CD11b+ NK1.1+. Macrophage 1 and 2 were differentiated by MHC2 + F4 80+ for M1 and by MHC2-F4 80+ for M2. The absolute cell counts in lung tissues for (**B**) M1, M2, (**C**) cDC1, cDC2, and NK cells were plotted by box; the line in the box represents the median cell amount (n = 4, except for the saline group on day 7 P.I., where n = 1 was used). Differences between groups were determined by a non-parametric comparison test, with *p*-values indicated as ** *p* < 0.01.

A total of 190,942 lung single cells per group on day 2 P.I. and a total of 179,842 lung single cells on day 7 P.I. were analyzed for macrophages, dendritic cells, NK cells, and gating strategies, as depicted in Figure 3A. Macrophages and conventional dendritic cells (cDCs) were divided into macrophage 1 (M1) and macrophage 2 (M2), and cDC1 and cDC2. M1 cells showed the highest cell amounts in the C_sd group on day 2 P.I., with a statistically significant difference compared to those in the E_hd group (*p* = 0.0053) (Figure 3B and Figure S2).

For dendritic cells, the E_hd group exhibited the highest cell amounts on day 2 P.I. for both cDC1 and cDC2, but no significant differences were observed. The C_hd group showed the highest cell amounts on day 2 P.I. for macrophages, dendritic cells, and NK cells, which is the same trend as in the T cell responses, but there were no statistically significant differences between groups (Figure 3B,C and Figure S2). NK cells showed a stronger presence in the saline group on day 2 P.I., and on day 7 P.I., the C_hd group had the highest expression, followed by the saline group. However, similar to the findings with the cDCs groups, there were no statistically significant differences between the groups (Figure 3B and Figure S2).

### 3.4. On Day 2 Post-Infection, the C_sd Group Showed High IFN-γ and TNF-α Secretion

**Figure 4:**
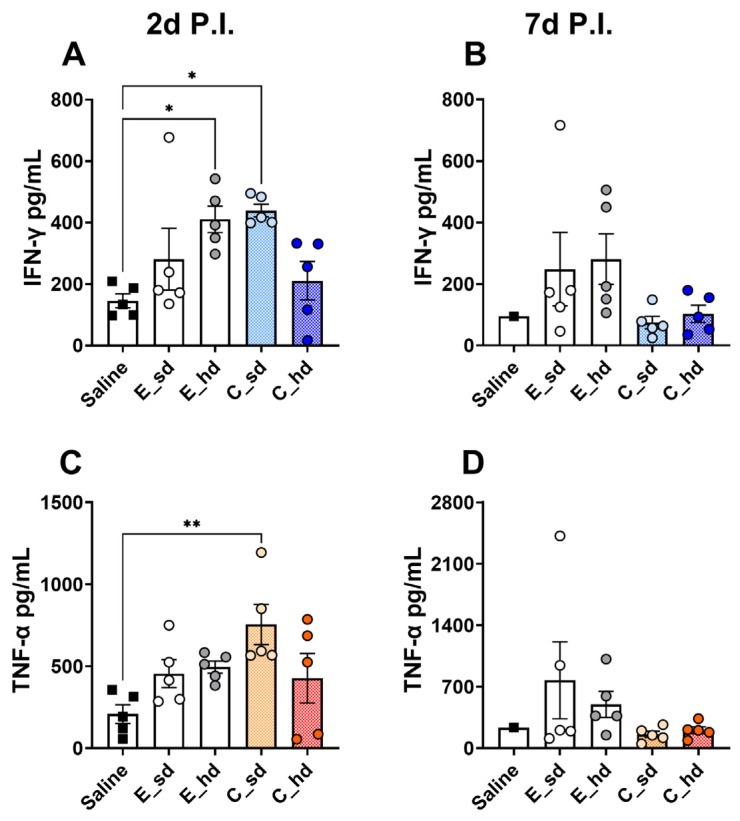
Cytokine response. Single lung cells were cultured for 72 h at 2 days and 7 days post-infection. After culturing, the supernatant was collected, and cytokine ELISA kits (Proteintech) were used to measure the levels of IFN-γ and TNF-α for each group (n = 5, with n = 1 for the saline group on day 7 P.I.). (**A**) IFN-γ secretion on day 2 P.I.; (**B**) IFN-γ secretion on day 7 P.I.; (**C**) TNF-α secretion on day 2 P.I.; (**D**) TNF-α secretion on day 7 P.I. All results are presented as the mean ± standard error of the mean (SEM). Statistical differences were analyzed using one-way ANOVA. *p*-values are indicated as follows: * *p* < 0.05, ** *p* < 0.01.

Regarding the inflammatory cytokines IFN-γ and TNF-α, the C_sd group showed the highest levels on day 2 P.I., which was consistent with the flow cytometry results (Figure 2A,D and Figure 4A,C). However, on day 7 P.I., the egg-cultivated vaccine groups exhibited higher levels, although there were no statistically significant differences between the groups (Figure 4B,D).

### 3.5. The E_hd Group Showed over 40 HAI Titers Against Four 2022–2023 Seasonal Flu Virus Strains

**Figure 5:**
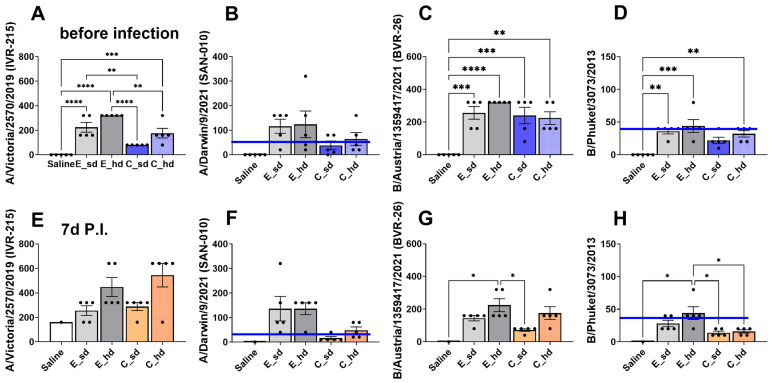
HAI titers. The HAI titer was measured at 2 weeks after the last of 2 intramuscular injections of the 2022–2023 Northern Hemisphere quadrivalent influenza vaccine, before infection. The HAI titer against (**A**) A/Victoria/2570/2019 (IVR-215) (A/H1N1), (**B**) A/Darwin/9/2021 (SAN-010) antigen (A/H3N2), (**C**) B/Austria/1359417/2021 (BVR-26) (B Victoria lineage), and (**D**) B/Phuket/3073/2013 (B Yamagata lineage) are shown with bar graphs. One week after an intranasal challenge with the A/Victoria/2570/2019 (IVR-215) (A/H1N1) strain, which occurred 3 weeks after the last vaccination (P.I. 7d), HAI titers were measured again. The HAI titer against (**E**) A/Victoria/2570/2019 (IVR-215) (H1N1), (**F**) A/Darwin/9/2021 (SAN-010) antigen (A/H3N2), (**G**) B/Austria/1359417/2021 (BVR-26) (B Victoria lineage), and (**H**) B/Phuket/3073/2013 (B Yamagata lineage) are shown with bar graphs. The blue line indicates the HAI titer threshold of 40. Serum from 5 mice per group was used, and the results were presented as mean HAI titer ± SEM (standard error of the mean) (for the saline group at P.I. 7d, n = 1 was used). Differences between groups were determined by one-way ANOVA, with *p*-values indicated as * *p* < 0.05, ** *p* < 0.01, *** *p* < 0.001, and **** *p* < 0.0001.

The C_sd group showed HAI titers below 40 for two strains, both after vaccination and after the challenge (Figure 5B,D,F,H). This was observed for antibodies against the A/Darwin/9/2021 (SAN-010) (H3N2) and the B/Phuket/3073/2013 (B Yamagata) strain. Particularly for the B/Phuket/3073/2013 (B Yamagata) strain, all vaccinated groups, except for the E_hd group, showed titers below 40 (Figure 5D,H). The E_hd group was the only group to display HAI titers above 40 for all strains and exhibited the highest HAI titers for all strains, both after vaccination and 1 week after the challenge (Figure 5A–H). However, in the A/Victoria/2570/2019 (IVR-215) (H1N1) strain, the C_hd group showed the highest average HAI titer of 735.2, surpassing the E_hd group and all other groups (Figure 5E).

The C_sd group had a pre-challenge HAI titer of 80, which increased to 278.6 at 1 week after the challenge with A/Victoria/2570/2019 (IVR-215) (H1N1), indicating more than a threefold increase. The C_hd group showed an even greater improvement, with its HAI titer rising from 160 pre-challenge to 735.2 post-challenge, more than a fourfold increase, indicating a significant boost in anti-HA antibodies following homologous virus infection (Figure 5E). The E_sd and E_hd groups, on the other hand, had sufficiently high pre-challenge HAI titers of 211.1 and 320, respectively, which increased to 242.5 and 422.2 post-challenge, but these titers did not show more than a twofold increase after the challenge (Figure 5E). For A/Victoria/2570/2019 (IVR-215) (H1N1), the C_sd group had the lowest average titer of 80 HAI before infection, showing a significant difference compared to the E_sd and E_hd groups. The C_hd group had an average titer of 160 HAI, which was statistically different from the E_hd group before infection. The C_sd and C_hd groups demonstrated significant differences, but both groups still showed a titer above 40 (Figure 5A).

## 4. Discussion

Traditional egg-based vaccines face several challenges, including prolonged production timelines, adaptive mutations, and allergy risks. In terms of mutations, it is important to note that pandemic-causing viruses have not exclusively originated from humans [21]. Specifically, avian viruses may have a higher likelihood of mutating during egg-based cultivation as they adapt to the host environment. This adaptation could increase the virus’s infectivity, particularly when it transitions to human-to-human transmission. Further research is needed to evaluate whether such mutations or egg-derived substances influence immunity positively or negatively [22].

This study evaluated the immunogenicity of standard-dose and high-dose cell-cultured (C_sd, C_hd) influenza vaccines versus egg-based (E_sd, E_hd) influenza vaccines in BALB/c mice challenged with the homologous A/Victoria/2570/2019 (H1N1) strain. Numerous studies have compared the immunogenicity of cell-based influenza vaccines to egg-based vaccines, indicating that cell-based vaccines are generally more effective across various age groups, including the elderly, children, and adolescents [23,24,25]. However, a review of multiple clinical trials and retrospective studies over the past decade reveals mixed evidence on whether cell-based vaccines consistently outperform egg-based vaccines [12]. Despite these mixed results and the higher cost of cell-cultured vaccines, experts argue that in real-world settings—where influenza leads to secondary complications such as hospital visits and deaths—cell-based vaccines are often more effective than egg-based options [14,26]. 

However, most of the studies comparing existing cell-based and egg-based influenza vaccines have focused on humoral responses or cohort studies and there are not an adequate number of comparative studies focusing on T cell responses. The significant role of T cells in influenza immunogenicity, including broad immunity, continues to be supported by many studies [27,28,29,30]. In particular, a recent cohort study using 965 samples confirmed that CD4+ T cells play an important role in influenza immunity and have cross-strain immunity, which is independent of HAI titers [31]. A previous comparison study between cell-derived influenza vaccine and egg-based vaccines [32] showed high T cell responses with a cell-cultured vaccine, which is confirmed by this study. 

The cell-cultured vaccine group had significantly higher IFN-γ+ CD8+ T cell, IFN-γ+ CD4+ T cell, CD4+ T_RM_ cell, and CD8+ T_RM_ cell responses than the egg-based vaccine group. T cells, particularly T_RM_ cells, also play a pivotal role in the duration of immunity. Previous studies have shown that although the number of T_RM_ cells in the lungs decreases 63 days after a viral infection, effective immunity can be sustained with continuous antigenic stimulation, specific cytokine signaling, and low-oxygen pressure environments [33,34]. Meanwhile, HA-neutralizing antibodies typically show a decline in titers around 6–7 months post-vaccination, though titers may persist for up to 12 months [35,36]. These results may support the opinion of experts that cell-grown vaccines would be more advantageous in the real world.

Neutralizing antibodies contribute to immunity during the initial stages of influenza virus infection. Alongside T cells, they play a crucial role in influenza immunity, as demonstrated in mouse models, where H7-specific neutralizing antibodies can protect against H7N9 infection [34]. In this study, the egg-based vaccine group showed equivalent vaccine efficacy in survival, body weight change, and lung viral titers compared to the cell-based group. We assume that the HAI titer against the homologous A/Victoria/2570/2019 (IVR-215) (H1N1) strain before the virus challenge influenced initial immunity [37,38] in the egg-based group. Nevertheless, other previous comparative studies have shown that cell-grown vaccines are not inferior to egg-based vaccines in HAI titers [12,39,40].

Alveolar macrophages have several important roles in lung tissue [41]. They are the first responders in influenza infection, binding to the influenza virus and preventing its spread through macrophage-mediated engulfment [42,43]. Macrophage 1(M1) bridges innate and adaptive immunity by presenting viral antigens to T cells, triggering an inflammatory response [44] and regulating the response. In this study, the C_sd group, which received a standard dose of cell-cultured vaccine, exhibited significantly higher levels of M1 on day 2 post-infection (P.I.) compared to the E_sd group. The similar expression trends of M1 and IFN-γ+ T cells suggest a strong interaction between these immune cells in the C_sd group, potentially triggering a broader interferon-stimulated gene (ISG) response. In a previous study by Jiang et al. [45], they demonstrated lung CD8+ T_RM_ cells produce immunity dependent on IFN-γ signaling, which is important to antiviral ISGs during the early stages of infection. Notably, we expect that our result of similar trends in M1, IFN-γ+ T cells, and T_RM_ cells on day 2 post-infection could support the result by Jiang et al. [45], and M1 could be one of the key immune cells for connecting these triggering immune responses.

Despite the high initial activation of these immune cells, the C_sd group showed the highest body weight loss among the vaccinated groups on day 3 P.I. This could be the result of an activated inflammatory reaction, but the C_sd group recovered the weight loss and showed the lowest viral load on day 7 P.I., indicating that the immune response was effectively regulated by the M2 response on day 7 P.I. and did not result in excessive inflammation [46,47]. Dendritic cells, particularly conventional dendritic cell 2 (cDC2), play a role in supporting humoral immune responses by activating T follicular helper cells. In this study, cDC2 expression was highest in the E_hd group on day 2 P.I. and in the C_hd group on day 7 P.I., but there were no significant differences between the vaccinated groups. The direct role of DCs in influencing the humoral response remains unclear [48]. 

The different level of T cell responses could be due to the potential sequence differences between the A/Victoria/2570/2019 IVR-215 (A/H1N1) strain and the vaccine strain, as exact matching is important for antigen presentation to T cells [49]. There was a study about poor cytokine-secreting T cell responses in the 2014–2015 influenza season when the antigenic drift of the A/H3N2 strain occurred [22]. Addressing the sequencing of commercial inactivated vaccines is a further challenge when performing whole vaccine sequencing for all strains. Moreover, the absence of antigen-specific in vitro stimulation in our assays restricts our ability to confirm that the T cell responses we measured are solely related to influenza. Residual host-cell proteins, lipid content, or egg-derived proteins may have contributed to or hampered the immune response. This study has other limitations, such as only challenging with the A/H1N1 strain, which should be considered. Also, a variety of cell-cultured and egg-based influenza vaccines are in use worldwide, and results may vary depending on individual vaccine characteristics rather than on whether they are cell-based or egg-based. We confirm comparable immunogenicity for cell- or egg-grown vaccines in domestic settings. Another limitation is the small sample size, especially in the saline-injected control group, which had only one mouse evaluated 7 days after the challenge. In addition, the exclusive use of BALB/c mice as the animal model may not fully represent the immune responses observed in humans, thereby limiting the applicability of the findings to broader populations. To fully understand the efficacy of the vaccines, further studies involving human participants are necessary.

## 5. Conclusions

A standard dose of a cell-cultured vaccine was found to significantly enhance the expression of IFN-γ+ T cells, T_RM_ cells, and M1 compared to an egg-based vaccine. Overall, the results suggest that cell-cultured vaccines may hold potential advantages for long-term immunity and high T cell responses, although further research in the real world is necessary. Both IFN-γ+ T cells and humoral immunity were shown to play equal roles in protection against influenza, as evidenced by the equivalent efficacy of the standard-dose egg-based vaccine; however, for a rapid pandemic response and to minimize risks associated with egg protein reactions and adaptive mutations, cell-cultured vaccines offer distinct benefits.

## Figures and Tables

**Table 1:** List of fluorescence antibodies used in T cell and immune cell staining.

**T cells**			
Manufacturer	Cat No.	Marker	Fluorescence
BD	564997	Live/dead	APC-R700 (Alexa Fluor 700)
BD	561100	CD3	PE-Cy7
Invitrogen	MCD0417	CD4	PE-Texas red
Invitrogen	35-5773-82	Foxp3	PE-Cy5.5
Invitrogen	15-0193-82	CD19	PE-Cy5
BD	563970	CD44	BV421_DAPI
Invitrogen	11-0621-82	CD62L	FITC
BD	563290	CD69	BV605
BD	557495	CD103	PE
BD	566409	CD8	BB700_PerCP Cy 5.5
Invitrogen	17-7177-81	IL-17	APC
Invitrogen	11-7311-82	IFN-γ	FITC
**Immune cells**			
Manufacturer	Cat No.	Marker	Fluorescence
Biolegend	423105	Live/dead	APC Cy7
Invitrogen	406-0031-82	CD3	BV605
Biolegend	103138	CD45	Amcyan
Biolegend	127615	Ly6G	PerCP Cy5.5
Invitrogen	35-0114-82	CD11c	PECy 5.5
Biolegend	101222	CD11b	Alexa 700
Biolegend	107607	MHC2	PE
Invitrogen	MF48017	F4 80	PE Texas Red
Invitrogen	17-5941-82	NK1.1	APC

**Table 2:** The 2022–2023 Northern Hemisphere strains used in the HAI assay.

Strains	NIBSC Code
A/Victoria/2570/2019 (IVR-215) (H1N1)	22/100
A/Darwin/9/2021 (SAN-010) antigen (H3N2)	21/320
B/Austria/1359417/2021 (BVR-26) (B Victoria lineage)	21/316
B/Phuket/3073/2013 (B Yamagata lineage)	21/136

## Data Availability

The datasets used and/or analyzed during the current study are available from the corresponding author upon reasonable request.

## Supplementary Materials

The following supporting information can be downloaded at https://www.mdpi.com/article/10.3390/vaccines12121392/s1, Figure S1: Dot plots for IL-17+ T cells. Figure S2: Dot plots for macrophages, dendritic cells and NK cells.

## Abbreviations

C_hd: cell-cultured high dose; C_sd: cell-cultured standard dose; E_hd: egg-based standard dose; E_sd: egg-based standard dose; HA: hemagglutinin; HAI: hemagglutinin inhibition assay; P.I.: post-infection; SEM: standard error of the mean; T_RM_: tissue-resident memory T cell.

## References

[1] Dawood F.S., Iuliano A.D., Reed C., Meltzer M.I., Shay D.K., Cheng P.-Y., Bandaranayake D., Breiman R.F., Brooks W.A., Buchy P. (2012). Estimated global mortality associated with the first 12 months of 2009 pandemic influenza A H1N1 virus circulation: A modelling study. Lancet Infect. Dis..

[2] Morens D.M., Taubenberger J.K., Harvey H.A., Memoli M.J. (2010). The 1918 influenza pandemic: Lessons for 2009 and the future. Crit. Care Med..

[3] Carter R.W., Sanford J.C. (2012). A new look at an old virus: Patterns of mutation accumulation in the human H1N1 influenza virus since 1918. Theor. Biol. Med. Model..

[4] Conrad A., Valour F., Vanhems P. (2023). Burden of influenza in the elderly: A narrative review. Curr. Opin. Infect. Dis..

[5] Molinari N.-A.M., Ortega-Sanchez I.R., Messonnier M.L., Thompson W.W., Wortley P.M., Weintraub E., Bridges C.B. (2007). The annual impact of seasonal influenza in the US: Measuring disease burden and costs. Vaccine.

[6] Wolf R.M., Antoon J.W. (2023). Influenza in Children and Adolescents: Epidemiology, Management, and Prevention. Pediatr. Rev..

[7] Orrico-Sánchez A., Valls-Arévalo Á., Garcés-Sánchez M., Álvarez Aldeán J., Ortiz de Lejarazu Leonardo R. (2023). Efficacy and effectiveness of influenza vaccination in healthy children. A review of current evidence. Enferm. Infecc. Microbiol. Clin. (Engl. Ed.).

[8] Feng S.Z., Jiao P.R., Qi W.B., Fan H.Y., Liao M. (2011). Development and strategies of cell-culture technology for influenza vaccine. Appl. Microbiol. Biotechnol..

[9] How Influenza (Flu) Vaccines Are Made. Centers for Disease Control and Prevention National Center for Immunization and Respiratory Diseases (NCIRD). https://www.cdc.gov/flu/vaccine-process/?CDC_AAref_Val.

[10] Robertson J.S., Bootman J.S., Newman R., Oxford J.S., Daniels R.S., Webster R.G., Schild G.C. (1987). Structural changes in the haemagglutinin which accompany egg adaptation of an influenza A(H1N1) virus. Virology.

[11] Zost S.J., Parkhouse K., Gumina M.E., Kim K., Diaz Perez S., Wilson P.C., Treanor J.J., Sant A.J., Cobey S., Hensley S.E. (2017). Contemporary H3N2 influenza viruses have a glycosylation site that alters binding of antibodies elicited by egg-adapted vaccine strains. Proc. Natl. Acad. Sci. USA.

[12] Álvarez Aldeán J., Salamanca I., Ocaña D., Barranco J.L., Walter S. (2022). Effectiveness of cell culture-based influenza vaccines compared with egg-based vaccines: What does the literature say?. Rev. Esp. Quimioter..

[13] Frey S., Vesikari T., Szymczakiewicz-Multanowska A., Lattanzi M., Izu A., Groth N., Holmes S. (2010). Clinical efficacy of cell culture–derived and egg-derived inactivated subunit influenza vaccines in healthy adults. Clin. Infect. Dis..

[14] Chi C., Cheng M., Ko K., Mould J.F., Chen C., Huang Y., Lee P. (2024). Cost-effectiveness analysis of cell-based versus egg-based quadrivalent influenza vaccines in the pediatric population in Taiwan. J. Med. Virol..

[15] Naleway A.L., Kim S.S., Flannery B., Levine M.Z., Murthy K., Sambhara S., Gangappa S., Edwards L.J., Ball S., Grant L. (2023). Immunogenicity of High-Dose Egg-Based, Recombinant, and Cell Culture-Based Influenza Vaccines Compared with Standard-Dose Egg-Based Influenza Vaccine Among Health Care Personnel Aged 18–65 Years in 2019–2020. Open Forum Infect. Dis..

[16] Lee L.Y.-H., Ha D.L.A., Simmons C., de Jong M.D., Chau N.V.V., Schumacher R., Peng Y.C., McMichael A.J., Farrar J.J., Smith G.L. (2008). Memory T cells established by seasonal human influenza A infection cross-react with avian influenza A (H5N1) in healthy individuals. J. Clin. Investig..

[17] Richards K.A., Topham D., Chaves F.A., Sant A.J. (2010). Cutting edge: CD4 T cells generated from encounter with seasonal influenza viruses and vaccines have broad protein specificity and can directly recognize naturally generated epitopes derived from the live pandemic H1N1 virus. J. Immunol..

[18] Zens K.D., Farber D.L. (2015). Memory CD4 T cells in influenza. Curr. Top. Microbiol. Immunol..

[19] McGee C.E., Sample C.J., Kilburg-Basnyat B., Gabor K.A., Fessler M.B., Gowdy K.M. (2019). Influenza-Mediated Lung Infection Models. Methods Mol. Biol..

[20] Broecker F., Liu S.T.H., Suntronwong N., Sun W., Bailey M.J., Nachbagauer R., Krammer F., Palese P. (2019). A mosaic hemagglutinin-based influenza virus vaccine candidate protects mice from challenge with divergent H3N2 strains. NPJ Vaccines.

[21] Konishi T. (2019). Re-evaluation of the evolution of influenza H1 viruses using direct PCA. Sci. Rep..

[22] Kim J.H., Mishina M., Chung J.R., Cole K.S., Nowalk M.P., Martin J.M., Spencer S., Flannery B., Zimmerman R.K., Sambhara S. (2016). Cell-Mediated Immunity Against Antigenically Drifted Influenza A(H3N2) Viruses in Children During a Vaccine Mismatch Season. J. Infect. Dis..

[23] Gärtner B.C., Beier D., Gosch G., Wahle K., Wendt L., Förster L.C., Schmidt K.J., Schwarz T.F. (2024). Cell-based influenza vaccines: An effective vaccine option for under 60-year-olds. GMS Hyg. Infect. Control.

[24] Mould-Quevedo J.F., Pelton S.I., Nguyen V.H. (2023). Vaccine Effectiveness of Cell-Based Quadrivalent Influenza Vaccine in Children: A Narrative Review. Vaccines.

[25] Stein A.N., Mills C.W., McGovern I., McDermott K.W., Dean A., Bogdanov A.N., Sullivan S.G., Haag M.D.M. (2024). Relative Vaccine Effectiveness of Cell- vs Egg-Based Quadrivalent Influenza Vaccine Against Test-Confirmed Influenza Over 3 Seasons Between 2017 and 2020 in the United States. Open Forum Infect. Dis..

[26] Nguyen V.H., Roy B. (2022). Modelling the Economic Impact of lnfluenza Vaccine Programs with the Cell-Based Quadrivalent Influenza Vaccine and Adjuvanted Trivalent Influenza Vaccine in Canada. Vaccines.

[27] Huang M., Xu R., Triffon C., Mifsud N., Chen W. (2021). Broad-Based Influenza-Specific CD8(+) T Cell Response without the Typical Immunodominance Hierarchy and Its Potential Implication. Viruses.

[28] Bodewes R., Kreijtz J.H., Hillaire M.L., Geelhoed-Mieras M.M., Fouchier R.A., Osterhaus A.D., Rimmelzwaan G.F. (2010). Vaccination with whole inactivated virus vaccine affects the induction of heterosubtypic immunity against influenza virus A/H5N1 and immunodominance of virus-specific CD8+ T-cell responses in mice. J. Gen. Virol..

[29] Sridhar S., Begom S., Bermingham A., Hoschler K., Adamson W., Carman W., Bean T., Barclay W., Deeks J.J., Lalvani A. (2013). Cellular immune correlates of protection against symptomatic pandemic influenza. Nat. Med..

[30] Dhume K., Finn C.M., Devarajan P., Singh A., Tejero J.D., Prokop E., Strutt T.M., Sell S., Swain S.L., McKinstry K.K. (2022). Bona Fide Th17 Cells without Th1 Functional Plasticity Protect against Influenza. J. Immunol..

[31] Tsang T.K., Lam K.-T., Liu Y., Fang V.J., Mu X., Leung N.H.L., Peiris J.S.M., Leung G.M., Cowling B.J., Tu W. (2022). Investigation of CD4 and CD8 T cell-mediated protection against influenza A virus in a cohort study. BMC Med..

[32] Brühl P., Kerschbaum A., Kistner O., Barrett N., Dorner F., Gerencer M. (2000). Humoral and cell-mediated immunity to vero cell-derived influenza vaccine. Vaccine.

[33] Martini V., Edmans M., Gubbins S., Jayaraman S., Paudyal B., Morgan S., McNee A., Morin T., Rijal P., Gerner W. (2022). Spatial, temporal and molecular dynamics of swine influenza virus-specific CD8 tissue resident memory T cells. Mucosal Immunol..

[34] Lim Y.S., Shin H. (2023). CD8 tissue-resident memory T cells bridge the gap between humoral and cell-mediated immunity. Mucosal Immunol..

[35] Young B., Sadarangani S., Jiang L., Wilder-Smith A., Chen M.I. (2018). Duration of Influenza Vaccine Effectiveness: A Systematic Review, Meta-analysis, and Meta-regression of Test-Negative Design Case-Control Studies. J. Infect. Dis..

[36] Kang K.R., Kim Y.J., Ahn M.B., Kang H.M., Kim S.K., Lee J.W., Chung N.G., Cho B., Jeong D.C., Kang J.H. (2022). Shorter duration of protection and lower geometric mean titers against A/H3N2 antigen of the quadrivalent influenza vaccine in children post-allogeneic hematopoietic stem cell transplantation. Bone Marrow Transpl..

[37] Li J., Zhang L., Bao L., Wang Y., Qiu L., Hu J., Tang R., Yu H., Shan J., Li Y. (2022). A broadly neutralizing human monoclonal antibody against the hemagglutinin of avian influenza virus H7N9. Chin. Med. J..

[38] Tomčíková K., Varečková E. (2019). Different mechanisms of the protection against influenza A infection mediated by broadly reactive HA2-specific antibodies. Acta Virol..

[39] Govorkova E.A., Kodihalli S., Alymova I.V., Fanget B., Webster R.G. (1999). Growth and immunogenicity of influenza viruses cultivated in Vero or MDCK cells and in embryonated chicken eggs. Dev. Biol. Stand..

[40] Reisinger K.S., Block S.L., Izu A., Groth N., Holmes S.J. (2009). Subunit influenza vaccines produced from cell culture or in embryonated chicken eggs: Comparison of safety, reactogenicity, and immunogenicity. J. Infect. Dis..

[41] Li H., Wang A., Zhang Y., Wei F. (2023). Diverse roles of lung macrophages in the immune response to influenza A virus. Front. Microbiol..

[42] Shiratsuchi A., Nakanishi Y. (2006). [Elimination of influenza virus-infected cells by phagocytosis]. Yakugaku Zasshi.

[43] Fujimoto I., Pan J., Takizawa T., Nakanishi Y. (2000). Virus clearance through apoptosis-dependent phagocytosis of influenza A virus-infected cells by macrophages. J. Virol..

[44] Meischel T., Villalon-Letelier F., Saunders P.M., Reading P.C., Londrigan S.L. (2020). Influenza A virus interactions with macrophages: Lessons from epithelial cells. Cell Microbiol..

[45] Jiang L., Liu L., Zhang M., Zhang L., Zhu C., He Q., Ye L., Zhao C., Li Z., Xu J. (2022). Prompt Antiviral Action of Pulmonary CD8+ T(RM) Cells Is Mediated by Rapid IFN-γ Induction and Its Downstream ISGs in the Lung. Front. Immunol..

[46] Hoagland D.A., Rodríguez-Morales P., Mann A.O., Yu S., Lai A., Vazquez A.B., Pope S.D., Lim J., Li S., Zhang S. (2023). Macrophages control pathological interferon responses during viral respiratory infection. bioRxiv.

[47] Petrich A., Dunsing V., Bobone S., Chiantia S. (2021). Influenza A M2 recruits M1 to the plasma membrane: A fluorescence fluctuation microscopy study. Biophys. J..

[48] Szulc-Dąbrowska L., Biernacka Z., Koper M., Struzik J., Gieryńska M., Schollenberger A., Lasocka I., Toka F.N. (2023). Differential Activation of Splenic cDC1 and cDC2 Cell Subsets following Poxvirus Infection of BALB/c and C57BL/6 Mice. Cells.

[49] Patel A., Kobinger G.P. (2012). Evaluation of Mismatched Immunity Against Influenza Viruses. Future Virol..

