# Supplementary material for: Cell-Cultured Influenza Vaccine Enhances IFN-γ+ T Cell and Memory T Cell Responses Following A/Victoria/2570/2019 IVR-215 (A/H1N1) Infection
Source: Vaccines (Basel). 2024 Dec 11;12(12):1392. doi: 10.3390/vaccines12121392 (PMC11680451; doi:10.3390/vaccines12121392)

Supplementary Figure S1.

(A) IL-17+ T cells were expressed as dot plots (n=5, with 1 mouse in the saline group on day 7 P.I.).

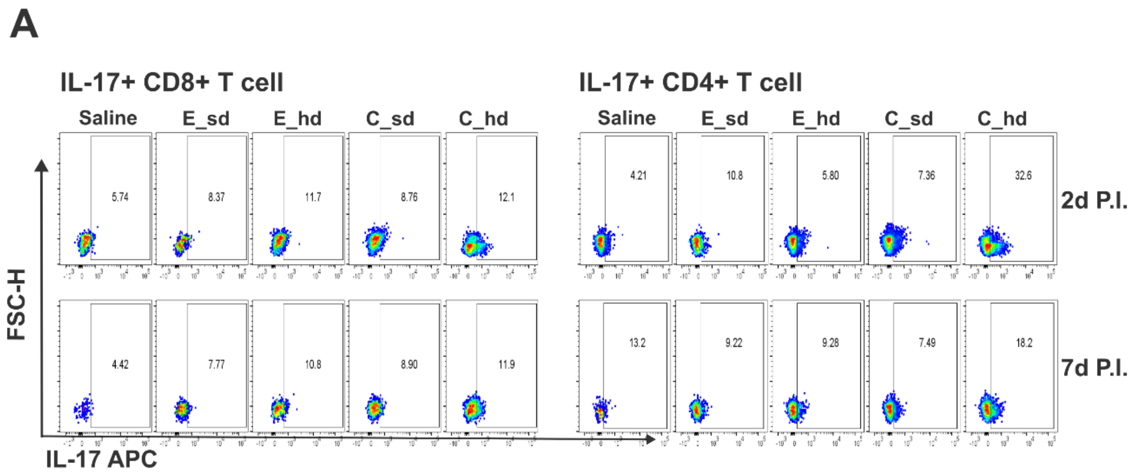

Supplementary Figure S2.

(A) Macrophage 1 (M1) and macrophage 2 (M2) (B) Conventional dendritic cell (cDC) 1, 2 and (C) NK cells were expressed as dot plots (n=4, with 1 mouse in the saline group on day 7 P.I.).

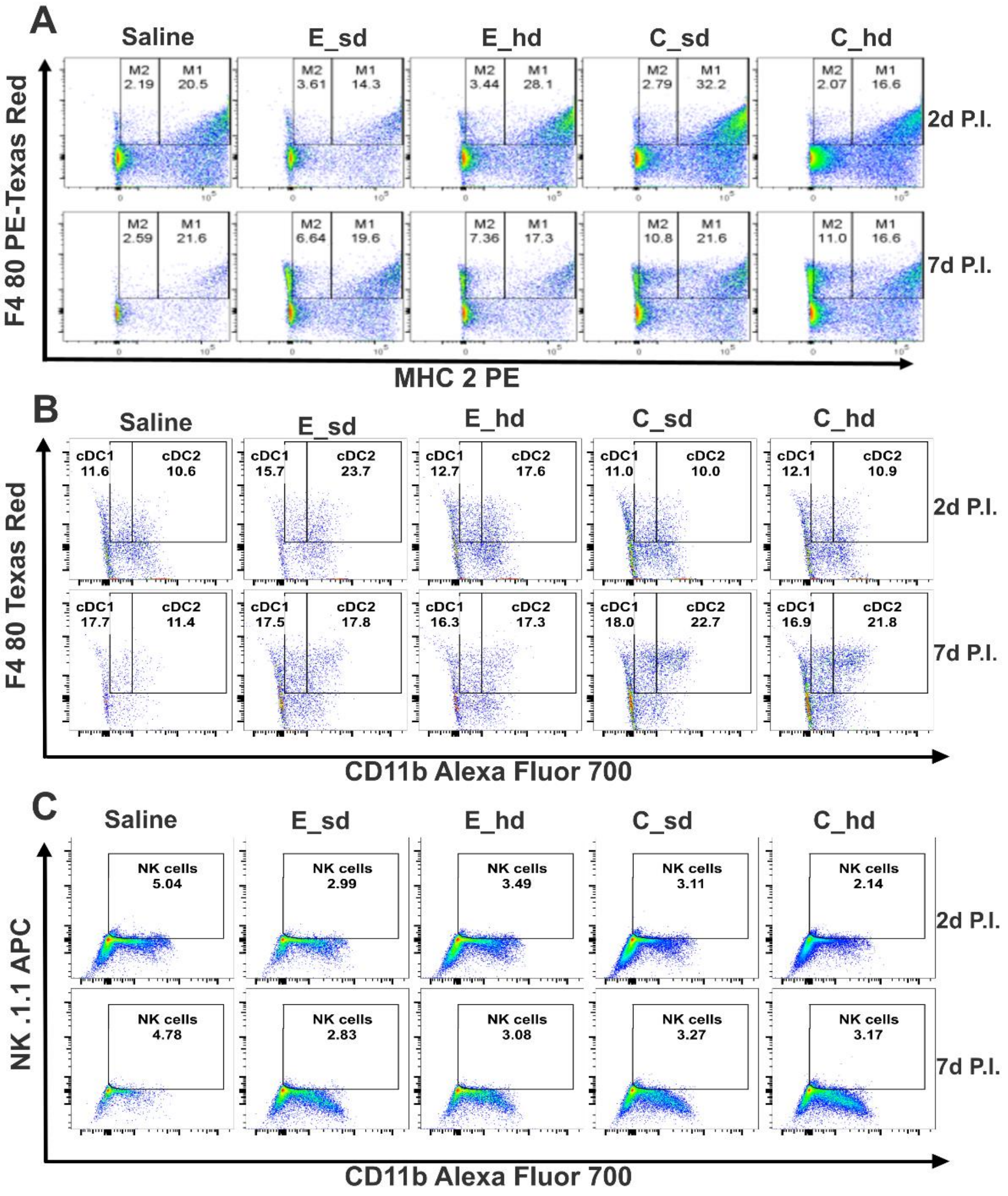

Supplement: Supplementary file 1 [file vaccines-12-01392-s001.zip › vaccines-3296145-supplementary.pdf]
